# Green synthesis of a typical chiral stationary phase of cellulose-tris(3, 5-dimethylphenylcarbamate)

**DOI:** 10.1186/1752-153X-7-129

**Published:** 2013-07-26

**Authors:** Run-Qiang Liu, Lian-yang Bai, Yi-Jun Zhang, Yu-Ping Zhang

**Affiliations:** 1 College of Plant Protection, Hunan Agricultural University, Changsha 410128, P.R. China; 2 Henan Institute of Science and Technology, Xinxiang 453003, P.R. China; 3 Hunan Academy of Agricultural Sciences, Changsha 410125, P.R. China

**Keywords:** Ionic liquid, AmimCl, HPLC, Chiral stationary phase, Pesticides, Chiral separation

## Abstract

**Background:**

At present, the study on the homogeneous-phase derivatization of cellulose in ionic liquid is mainly focused on its acetylation. To the best of our knowledge, there has been no such report on the preparation of cellulose-tris(3,5-dimethylphenylcarbamate) (CDMPC) with ionic liquid 1-allyl-3-methyl-imidazolium chloride (AmimCl) so far.

**Results:**

With ionic liquid 1-allyl-3-methylimidazolium chloride (AmimCl) as a reaction solvent, cellulose-tris(3,5-dimethylphenylcarbamate) (CDMPC) was synthesized by the reaction of 3,5-dimethylphenyl isocyanate and soluble microcrystalline cellulose in a homogeneous phase. The synthesized CDMPC was then coated onto the surfaces of aminopropyl silica gel to prepare a chiral stationary phase (CSP). The prepared CSP was successfully used in chiral separation of seven racemic pesticides by high performance liquid chromatography (HPLC). Good chiral separation was obtained using n-hexane and different modifiers as the mobile phases under the optimal percentage and column temperature, with the resolution of metalaxyl, diniconazole, flutriafol, paclobutrazol, hexaconazole, myclobutanil and hexythiazox of 1.73, 1.56, 1.26, 1.00, 1.18, 1.14 and 1.51, respectively. The experimental results suggested it was a good choice using a green solvent of AmimCl for cellulose functionalization.

**Conclusion:**

CDMPC was successfully synthesized as the chiral selector by reacting 3, 5-dimethylphenyl isocyanate with dissolved microcrystalline cellulose in a green ionic liquid of AmimCl.

## Findings

Enantioseparation is an effective approach in the determination of purity of enantiomers and preparation of a single enantiomer in the research fields, such as chiral drugs, natural products, and agricultural chemicals [1]. The key in the chiral technique is the design and application of chiral stationary phases (CSPs) [2-5]. At present, the polysaccharide derivatives, especially benzoate and phenylcarbamate derivatives of cellulose and starch, are most popular CSPs for direct separation of enantiomers with HPLC due to their strong chiral recognition capability [6-9]. Generally, the synthesis of cellulose benzoates started from cellulose suspension in pyridine and carried out heterogeneously. Ionic liquid is an organic salt in the form of liquid under normal temperature, which has attracted wide attention from the chemistry community for its advantages of low melting point, non-volatility, and adjustable structure and property, etc. [10-12]. Recently, Swatloski et al. found that 1-butyl-3-methylimidazolium chloride (BmimCl) ionic liquid was able to dissolve cellulose [13], which opened a new field for the study and development of cellulose solvent system. Wu et al., not only proved 1-allyl-3-methylimidazolium chloride (AmimCl) to be a good solvent for cellulose, but also successfully synthesized cellulose acetate in it [14]. At present, the study on the homogeneous-phase derivatization of cellulose in an ionic liquid is mainly focused on its acetylation. There has been no report on the esterification of cellulose in an ionic liquid of AmimCl so far.

In this study, CDMPC, as a chiral selector, was firstly prepared in an ionic liquid of AmimCl, followed by the characterization of FTIR, ^1^HNMR and elementary analysis. After it was coated on the surfaces of silica gel and packed into a chromatographic steel column, seven racemic pesticides including diniconazole, flutriafol, paclobutrazol, myclobutanil, hexaconazole, metalaxyl and hexythiazox, were selected for the chromatographic evaluation of enantioselective ability in normal- phase HPLC.

### Experiment

#### Equipments and reagents

Chromatographic measurements were made on a Agilent 1100 HPLC system (Agilent Technologies, Inc., Walbronn, Germany) equipped with a quaternary pump, a vacuum degasser module, a Rheodyne 7725i injector with a 20 μL sample loop, a temperature controlled column compartment and a variable wavelength UV detector. FTIR spectra were recorded in the range 400–4000 cm^−1^ with 4 cm^−1^ resolution with a BRUKER TENSOR27 system (Bruker Scientific Technology Co. Ltd., Karlsruhe, Germany). The 400 MHz 1H NMR spectra were measured on a BRUKER ARX 400 spectrometers (Bruker Scientific Technology Co. Ltd., Karlsruhe, Germany).

AmimCl was purchased from Shanghai Cheng Jie Chemical Co. Ltd. (Shanghai, China). 5 μm spherical silica gel (Kromasil, pore size 10 nm) was purchased from Akzo Noble N.V. (Nacka, Sweden). Microcrystalline cellulose (Avicel, DP = 200), tetrahydrofuran (THF), 3-aminopropyltriethoxysilane and 3, 5-dimethylphenyl isocyanate were purchased from Aladdin Chemistry Co. Ltd. (Shanghai, China). Diniconazole (97.4%), flutriafol (97%), paclobutrazol (97.4%) and myclobutanil (98%) were provided by Hunan Research Institute of Chemical Industry, hexaconazole (95%), metalaxyl (98%) and hexythiazox (98%)were provided by Hunan Institute for Food and Drug Control.

#### Synthesis of CDMPC in AmimCl

Synthesis of CDMPC was carried out according to the previous report [15], but the great difference was that an ionic liquid of AmimCl was used instead of traditional pyridine solvent.

**Figure 1:**
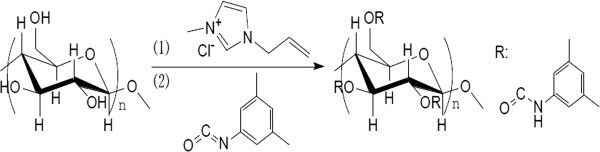
Synthesis of CDMPC in AmimCl.

As shown in Figure 1, 1.00 g of dry microcrystalline cellulose was transferred into a 100 ml three-neck flask, and the flask was added with 34.00 g of AmimCl (1). The solution was stirred magnetically for dissolution (90°C, 3 h). The flask was added with 10.00 mL of 3, 5-dimethylphenyl isocyanate (2), then the solution was refluxed for 48 h. The whole process was conducted under the protection of N_2_. After the solution was cooled down, it was added with 800 ml of methanol. The sediment was washed with 200 ml of methanol, and then dried for 12 h under vacuum at 80°C. 2.93 g of white solid of CDMPC was produced with a yield of 79.19%.

#### Preparation of CDMPC-coated CSP

CDMPC-coated CSP was prepared according to the published reference [16]. Firstly, 3.00 g of silica gel was transferred into toluene, and sufficiently reacted with 3- aminopropyltriethoxysilane at 110°C for 48 h to obtain aminopropyl-silica gel (APS). Secondly, 0.45 g of the synthesized CDMPC was weighed and dissolved in 30 mL of tetrahydrofuran. Then the solution was added to 2.55 g of APS, and dried for 8 h (60°C, vacuum condition) after the solvent was removed under vacuum at room temperature. A CDMPC-coated CSP with coating quantity of 15% was thus prepared.

#### Column packing

With isopropanol as slurry solvent and the mixture of n-hexane/isopropanol (90/10, v/v) as displacement fluid, the CDMPC-coated CSP was filled into the stainless-steel column (250 mm × 4.6 mm) under a pressure of 7000 psi.

### Results and discussion

#### Characterization of CDMPC

**Figure 2:**
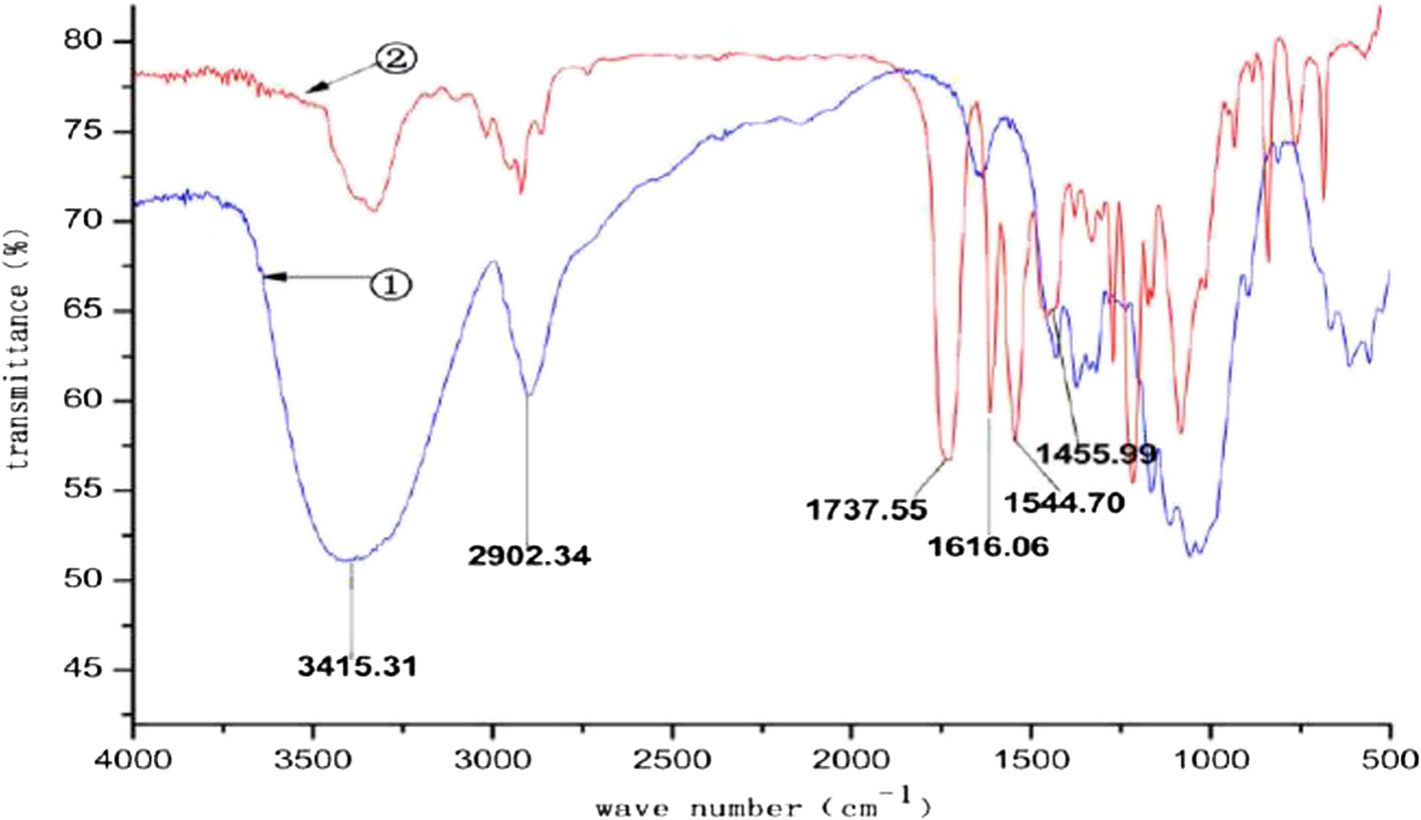
**FTIR spectra of cellulose and CDMPC.** ① Cellulose, ② CDMPC.

FTIR spectra of microcrystalline cellulose and CDMPC were compared in Figure 2. The spectrum of CDMPC had a significantly weaker -OH absorption peak at 3415.31 cm^-1^, suggesting that the -OH groups of cellulose were mostly replaced. Peak at 2902.34 cm^-1^ stands for the stretching vibration peak of -CH_3_ group, the absorption peaks of the benzene ring appeared at 1616.06 cm^-1^, 1544.70 cm^-1^ and 1455.99 cm^-1^, the absorption peak of the carbonyl group was found at 1737.55 cm^-1^. These all suggest that the -OH groups on microcrystalline cellulose have reacted with 3, 5-dimethylphenyl isocyanate.

**Figure 3:**
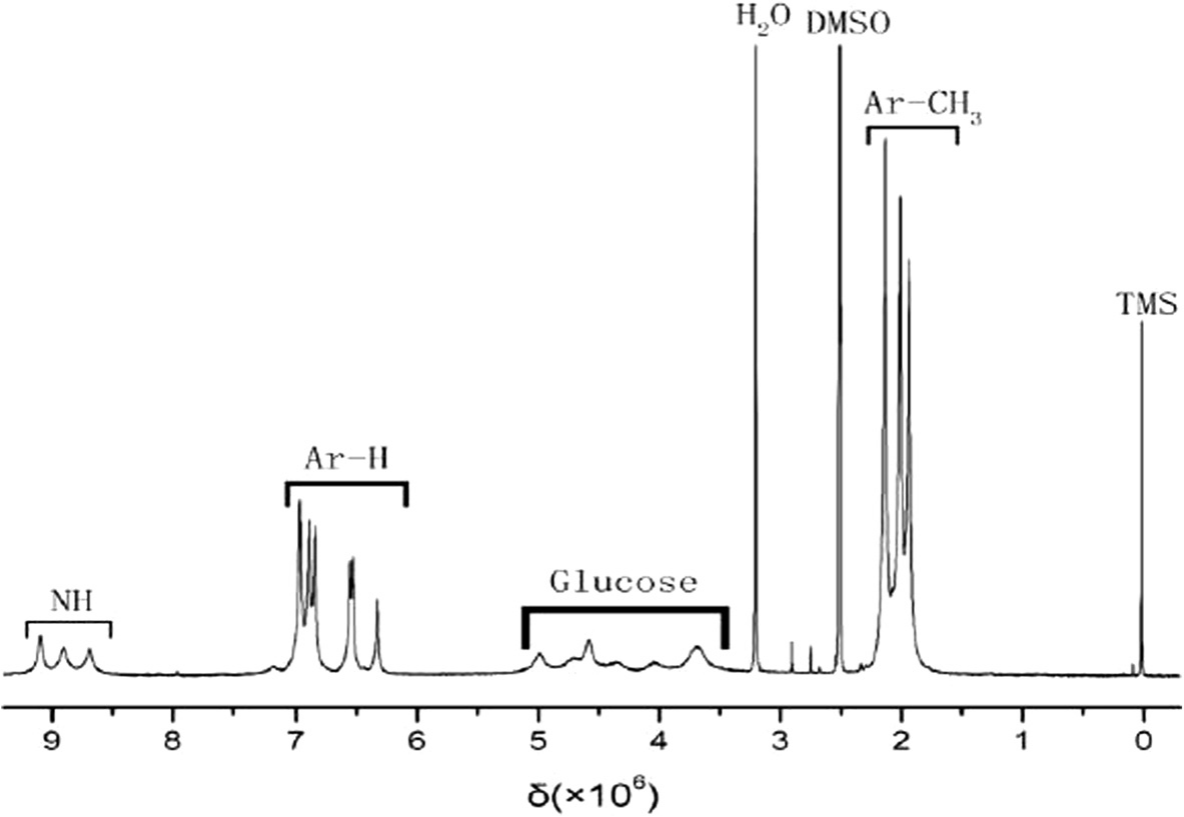
NMR spectrum of CDMPC.

The obtained CDMPC was also determined by the ^1^H NMR (400 MHz, 50°C, DMSO-d_6_). As shown in Figure 3, the chemical shifts (δ, ppm) could be observed at different positions of 8.5-8.9 (N-H), 6.3-7.1 (Ar-H), 3.5-5.1 (Glucose-H), 1.8-2.2 (Ar-CH_3_).

From the molecular formula of CDMPC (C_33_H_37_O_8_N_3_), its theoretical components of each element are C 65.70%, H 6.18% and N 6.96%, respectively. In contrast, the measured components of CDMPC by elemental analysis are C 64.40%, H 6.26% and N 6.73%. The comparable results showed no obvious difference of element content was found, suggesting that CDMPC was successfully synthesized by the developed method.

#### Chromatographic evaluation for the coated CSPs

**Figure 4:**
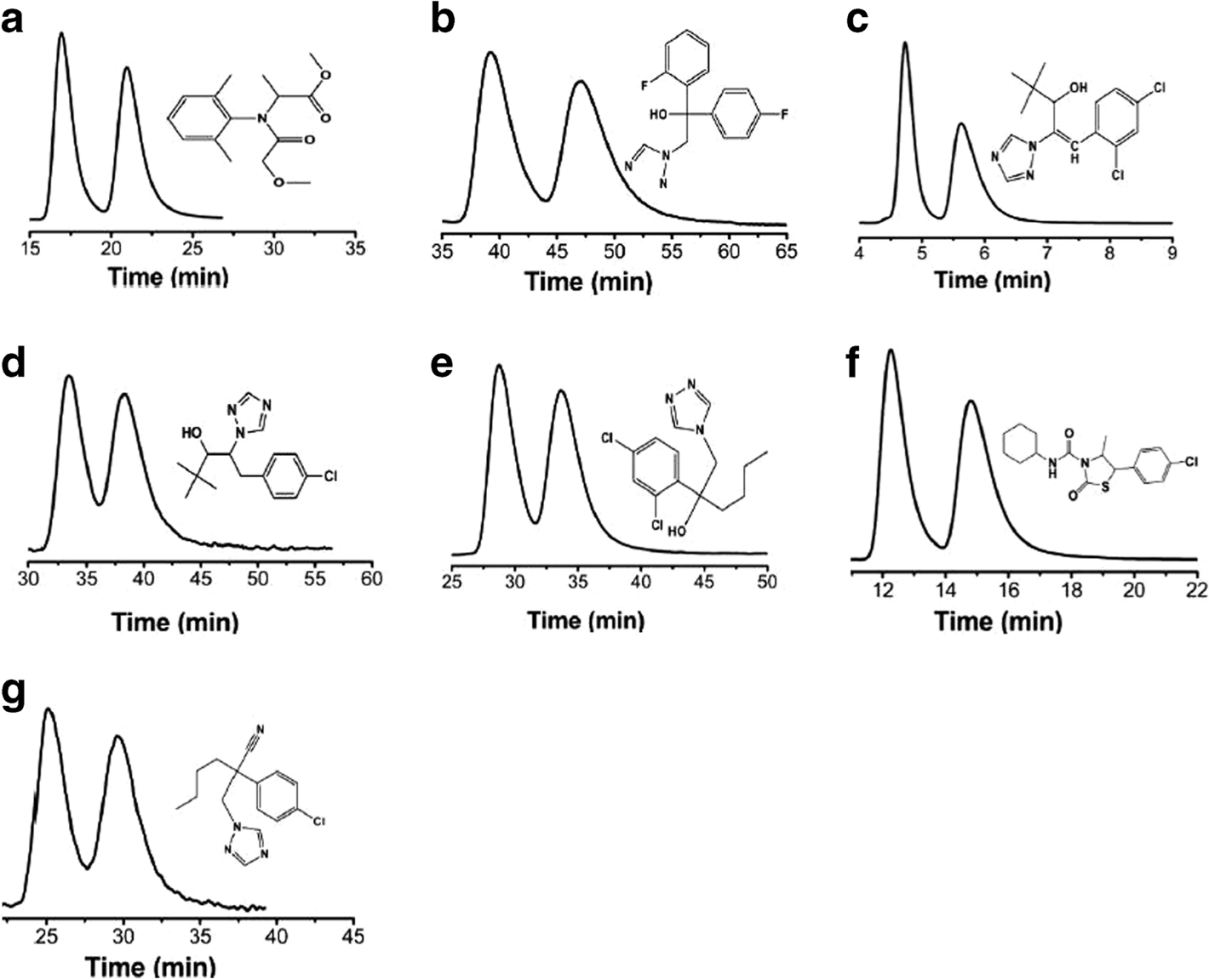
**Optimal chromatograms of the selected pesticides and their molecular structures.** Pesticide identification: **(a)** metalaxyl, **(b)** flutriafol, **(c)** diniconazole, **(d)** paclobutrazol, **(e)** hexaconazole, **(f)** hexythiazox, **(g)** myclobutanil.

Diniconazole, flutriafol, paclobutrazol and hexaconazole all contain benzene rings and hydroxyls in their molecular structures, hexythiazox and metalaxyl contain benzene rings and carbonyls in their molecular structures, myclobutanil, diniconazole, paclobutrazol and hexaconazole contain chlorphenyls and 1, 2, 4-triazolyls in their molecules. The above groups may have dipole-dipole interaction, hydrogen bond interaction and π-π interaction with the stationary phases. Generally, the polarity of mobile phase can be changed by changing the alcohol ratio in mobile phase, which in turn affects the retention time and resolution in enantioseparation [17]. In order to preliminarily study the separation effects of seven racemic pesticides and investigate the impact of polarity on separation, we selected isopropanol as the polar modifier. The impact of modifier (isopropanol/ethanol) and separation temperature on the retention time and stereo selectivity of enantiomer separation of 7 racemic pesticides on the self-made CDMPC chiral column were investigated in detail. When n-hexane/isopropanol (98/2, v/v) were used as the mobile phases with a flow rate of 1.0 ml/min at 25°C, the resolution of paclobutrazol, hexaconazole and hexythiazox was 1.00, 1.18 and 1.51, respectively, the resolution of diniconazole was 1.56 when n-hexane/isopropanol (80/20, v/v) were used as the mobile phases at 25°C, the resolution of flutriafol was 1.26 when n-hexane/isopropanol (95/5, v/v) were used as the mobile phases at 20°C, the resolution of myclobutanil was 1.14 when n-hexane/isopropanol (85/15, v/v) were used as the mobile phases at 25°C, the resolution of metalaxyl was 1.73 when n-hexane/ethanol (95/5, v/v) were used as the mobile phases at 25°C. The optimal separation chromatograms of 7 racemic pesticides were shown in Table 1 and Figure 4.

**Table 1:** The resolutions under optimized conditions

**Sample**	**Optimized condition**	***k1***	***k2***	**α**	**Rs**
Paclobutrazol	25°C, 2% isopropanol in *n-*hexane, 1.0 mL · min^-1^, 222 nm	*9.93*	*11.52*	1.16	1.00
Hexaconazole	25°C, 2% isopropanol in *n-*hexane, 1.0 mL · min^-1^, 230 nm	8.81	10.48	1.19	1.18
Hexythiazox	25°C, 2% isopropanol in *n-*hexane, 1.0 mL · min^-1^, 230 nm	*3.10*	*3.95*	1.27	1.51
Diniconazole	25°C, 20% isopropanol in *n-*hexane, 1.0 mL · min^-1^, 230 nm	0.60	0.91	1.52	1.56
Flutriafol	5°C, 5% isopropanol in *n-*hexane, 1.0 mL · min^-1^, 214 nm	15.16	19.11	1.26	1.26
Myclobutanil	25°C, 15% isopropanol in *n-*hexane, 1.0 mL · min^-1^, 210 nm	7.48	8.98	1.20	1.14
Metalaxyl	25°C, 5% ethanol in *n-*hexane, 1.0 mL · min^-1^, 214 nm	4.75	6.11	1.29	1.73

The direct enantioseparation of seven pesticides has been achieved, demonstrating the high chiral recognition abilities for the coated CDMPC in chiral HPLC. Good chiral separation was probably attributed to the hydrogen bond interaction, dipole-dipole interaction and π-π interaction between the CSPs and the recognized analyte [18].

### Conclusions

With the green solvent of AmimCl as a homogenous reaction medium, CDMPC was successfully prepared by the reaction of 3, 5-dimethylphenyl isocyanate with soluble microcrystalline cellulose. Its chemical structure was further proved by the FTIR, ^1^HNMR and elementary analysis. The prepared CDMPC-coated CSPs was successfully used in chiral separation of seven pesticides by HPLC. Seven racemic pesticides were well separated under the optimized chromatographic conditions. It suggested that it was a good alternative to synthesize the selector in the presence of a low-cost, environmentally friendly cellulose solvent.

## Description of additional material

The synthetic method of CDMPC by reacting 3, 5-dimethylphenyl isocyanate with dissolved microcrystalline cellulose in a green ionic liquid of AmimCl are available. The data characterization of FTIR, 1HNMR and elemental analysis are also available.

## Abbreviations

CDMPC: Cellulose-tris(3,5-dimethylphenylcarbamate); AmimCl: 1-allyl-3-methyl-imidazolium chloride; CSPs: Chiral stationary phase; HPLC: High performance liquid chromatography; THF: Tetrahydrofuran; APS: Aminopropyl-silica gel.

## Competing interests

The authors declare that they have no competing interests.

## Authors’ contributions

BLY and ZYP initiated and designed the study. All authors contributed to data analyses and to finalizing the manuscript. All authors have read and approved the final version.
